# Objective Neuropsychological Deficits in Post-Traumatic Stress Disorder and Mild Traumatic Brain Injury: What Remains Beyond Symptom Similarity?

**DOI:** 10.3390/bs4040471

**Published:** 2014-12-01

**Authors:** Hélène Pineau, André Marchand, Stéphane Guay

**Affiliations:** 1 Department of Psychology, Université du Québec à Montréal, Quebec, H2X 3P2, Canada; E-Mail: h.pino@hotmail.com; 2 Lucie-Bruneau Rehabilitation Centre, Montreal, Quebec, H2H 2N8, Canada; 3 Institut Universitaire en Santé Mental de Montréal, Montreal, Quebec, H1N 3M5, Canada; E-Mail: stephane.guay@umontreal.ca; 4 Department of Criminology, Université de Montréal, Montreal, Quebec, H3C 3J7, Canada

**Keywords:** neuropsychological deficits, PTSD, mTBI, comorbidity, symptom similarity

## Abstract

This exploratory study intends to characterize the neuropsychological profile in persons with post-traumatic stress disorder (PTSD) and mild traumatic brain injury (mTBI) using objective measures of cognitive performance. A neuropsychological battery of tests for attention, memory and executive functions was administered to four groups: PTSD (*n* = 25), mTBI (*n* = 19), subjects with two formal diagnoses: Post-traumatic Stress Disorder and Mild Traumatic Brain Injury (mTBI/PTSD) (*n* = 6) and controls (*n* = 25). Confounding variables, such as medical, developmental or neurological antecedents, were controlled and measures of co-morbid conditions, such as depression and anxiety, were considered. The PTSD and mTBI/PTSD groups reported more anxiety and depressive symptoms. They also presented more cognitive deficits than the mTBI group. Since the two PTSD groups differ in severity of PTSD symptoms but not in severity of depression and anxiety symptoms, the PTSD condition could not be considered as the unique factor affecting the results. The findings underline the importance of controlling for confounding medical and psychological co-morbidities in the evaluation and treatment of PTSD populations, especially when a concomitant mTBI is also suspected.

## 1. Introduction

### 1.1. Studies of Cognitive Problems in PTSD Populations

In one of the few literature reviews focusing on objective long-term memory problems in individuals with PTSD, Isaac and colleagues concluded that the memory problems reported in this population were primarily due to attention problems [1]. The authors found that deficits on span tasks were reported in participants in only three out of nine studies, whereas deficits on measures of complex attention (e.g., divided attention tasks) were observed in participants in seven of the nine studies included in the review. The participants’ problems manifested in verbal long-term memory seemed to depend on attentional resources recruited during memorization tasks. The majority of the 20 studies included in the review were conducted with very small samples of Vietnam veterans (*n* < 11) and less than half of the participants had actual diagnoses of PTSD at the time of the evaluation. With the exception of possible exposure to toxins, no exclusion criteria related to possible brain trauma, past or present health problems, developmental problems or substance abuse were mentioned.

In a sample of 21 refugees with or without PTSD, Johnsen, Kanagaratnam [2] attempted to control neurological comorbidity factors by excluding participants with “cerebral damage or neurological illness” or “suspected” brain trauma or alcohol abuse. The authors analyzed participants’ performances on a verbal long-term memory task, while controlling for comorbid depression, and found that depression explained the greatest amount of the variance in scores on the repeated recall task. The results demonstrate the importance of exerting strict control over possible confounding factors in studies of cognitive performance in PTSD populations.

### 1.2. Studies of Cognitive Problems in mTBI Populations

The Belanger, Curtiss [3] meta-analysis of cognitive problems in individuals with Mild Traumatic Brain Injury (mTBI) included 39 studies selected according to strict sampling criteria. The results revealed differences in cognitive profiles according to elapsed time since the trauma. The most common difficulties reported by individuals with acute mTBI (less than three months post-trauma) were related to verbal fluidity and differed recall of information in long-term verbal memory. In non-clinical samples, residual deficits on neuropsychological tests did not persist past three months post-trauma. In comparison, persistent cognitive sequelae were observed in clinical samples and in samples including individuals in litigation processes at three or more months post-trauma. However, Belanger, Curtiss [3] emphasized that some of the studies in their review failed to take certain important factors into account, thereby limiting the interpretation of the results. Factors not considered included subtle neurological sequelae (e.g., complex attention problems that are not perceptible on less sensitive neuropsychological tests, accentuation of previous personality traits and psychological factors associated with mTBI (e.g., emotional reactions associated with the trauma).

Bélanger *et al.*’s [3] results also highlighted the need to control for psychological/medical antecedents and comorbidity, elapsed time between the trauma and the evaluation, sample provenance and other factors that could affect results on cognitive tests.

### 1.3. Studies of Cognitive Problems in Individuals with Double Diagnoses (mTBI and PTSD)

Generally, similar symptoms in both diagnoses are complaints concerning difficulties of attention and memory, irritability, sleeping disorders as well as avoidance behaviors. Yet, if these symptoms seem to have a psychological origin, connected to the after-effects of post-traumatic stress disorder, some of these symptoms can also be the direct consequence of mTBI. Avoidance symptoms often reported by people with mTBI can be the result of neuropsychological after-effects of post-commotional cognitive disorders (e.g., difficulties following conversation among individuals with mTBI avoiding social situations) and not the consequence of anxiety activated by flashbacks of the traumatic event, as it is often the case with PTSD. Two studies [4,5] explored the nature of complaints in individuals with PTSD and self-reported mTBI. Hoge, McGurk [4] noted that the physical and cognitive complaints characteristic of mTBI (e.g., brief loss or alteration in consciousness) reported by veterans from Iraq are also characteristic of PTSD and comorbid depression. The authors inferred a mediating role of PTSD and depression in the expression of physical and cognitive symptoms in veterans with presumed double diagnoses. Vanderploeg, Belanger [5] studied a mixed group comprising soldiers and civilians with chronic PTSD and subsequent mTBI sustained several months or years later. Their results suggested that, when mTBI occurs in the context of pre-existing PTSD, the effectiveness of psychological treatment for the trauma symptoms is compromised. According to Vanderploeg, Belanger [5], PTSD contributes more significantly to subjective physical, cognitive and emotional symptoms than mTBI, and that neither condition moderates the symptomatology of the other; rather, their effect is cumulative.

Certain methodological problems in the previous studies limit the generalization of findings. For example, the conclusions of two previous studies [4,5] are respectively based on statistical analyses conducted with data based on a few general questions concerning subjective attention and memory complaints and gathered in clinical populations whose diagnoses were established from subjective, retrospective reports. Also, the diagnoses of mTBI and PTSD were not based on formal medical or psychological evaluation, and the absence of a control group precludes information about baseline symptoms in the general population. Finally, these studies failed to sufficiently control for the presence of pre-existing or concurrent medical, neurological, and developmental conditions, making it difficult to know if participants’ reports of their complaints were unaffected by these conditions.

In a study by Nelson, Yoash-Gantz [6], veterans of the Iraq and Afghanistan operations with a comorbid PTSD and mTBI completed attention tests. In comparison to participants with mild or moderate traumatic brain injury without PTSD, the participants with a comorbid PTSD and mTBI demonstrated deficits in response inhibition and significant slowing in information processing. However, certain methodological weaknesses limited the interpretation of the results. For example, the study used self-report questionnaires of symptoms to establish PTSD diagnoses, and mTBI diagnoses were exclusively based on the “veteran’s memory of the duration of the presumed loss of consciousness”. Further, participants were evaluated an average of 16 months after the injury, introducing the possibility of memory bias. Finally, participants’ medical history and psychiatric comorbidity were not controlled.

### 1.4. Objectives and Hypotheses

Is it possible to establish distinct neuropsychological profiles for individuals with mTBI, individuals with PTSD, and individuals with a comorbid mTBI and PTSD PTSD and mTBI, respectively, beyond their common symptoms? Using the results of objective tests to establish distinct profiles could facilitate the differentiation of symptoms during diagnosis and promote the development of specialized treatment plans for each group. This is the long-term objective of the current study. To meet our objective, we tried to avoid the methodological weaknesses of past studies and eliminate confounding variables.

*The hypotheses of the present study are:* (1) The PTSD group will demonstrate significantly greater deficits on measures of attention and memory than the control group (if PTSD alone impacts cognitive performance independently of mTBI); (2) Cognitive performance will be negatively correlated with PTSD severity; (3) The comorbid PTSD and mTBI group (mTBI/PTSD) will demonstrate significantly greater deficits in the cognitive tests than will the PTSD and mTBI groups (if the combination of the two conditions has a cumulative effect, as Vanderploeg’s hypothesis postulates).

## 2. Method

### 2.1. Inclusion Criteria

In total, 75 subjects were distributed through the study groups. The subsample sizes are specified in each subsection of the inclusion criteria

#### 2.1.1. PTSD Group

Twenty-five participants with formal diagnoses of PTSD were included in the study. The participants were recruited through advertisements in the community or were referred by medical or mental health professionals at the *Centre d’Étude du Trauma* (Centre for Trauma Research) at the Institut Universitaire en Santé Mentale de Montréal.

#### 2.1.2. mTBI Group

Nineteen participants with formal diagnoses of mTBI (based on medical records) were included in the present study. Criteria for mTBI were based on the international definition of mild traumatic brain injury (see Carroll, Cassidy [7]) and comprised the following: period of altered consciousness less than 30 min, Glasgow Coma Scale score between 13 and 15, and post-traumatic amnesia (PTA) for less than 24 h. Formal mTBI diagnoses was established by doctor (neurologist or emergency physician) following admission in emergency department or later, during the medical evaluation process of neurological sequelae (*See Table 1 for elapsed time since diagnosis for each clinical groups*). Participants were recruited at a tertiary rehabilitation centre for traumatic brain injury (*Centre de Réadaptation Lucie-Bruneau* (Lucie Bruneau Rehabilitation Center)) in Montreal.

#### 2.1.3. mTBI/PTSD Group

Following the evaluation of medical history and psychiatric condition, six of the 25 participants with mTBI were diagnosed with post-traumatic stress disorder following evaluation using the SCID-I (The Structured Clinical Interview for DSM-IV–for Axis I).

#### 2.1.4. Control Group

The twenty-five participants in this group were civilians recruited through newspaper or Internet advertisements. Each control participant was paired with a PTSD and/or mTBI participant, according to age, level of education, and gender.

### 2.2. Language and Litigation

The PTSD and mTBI groups were similar across ethnicity and spoken language (mostly Caucasian, with French as a first language). Nine participants in the PTSD group were involved in litigation and one was excluded from the study as the subject did not meet the diagnostic criteria during the evaluation process. None of the participants in the mTBI group were involved in litigation at the time of the evaluation.

### 2.3. Exclusion Criteria

Exclusion criteria for the present study were the following: (1) unstable medical conditions, past history of traumatic brain injury before the more recent one, or other diseases with the potential to affect brain functioning in pre- or post-trauma; (2) existing substance abuse problem; (3) learning disorder or attention deficit in school; (4) history of physical violence during infancy; and (5) an incapacitating physical disorder that is not adequately controlled; (6) a psychotic episode (past or present); (7) a bipolar disorder, or an organic mental disorder.

### 2.4. Procedure

Participation in the study was voluntary; all participants provided written consent prior to the study and were offered financial compensation for their time (Can$20.00 per session of evaluation). As a prerequisite for taking part in the study, participants underwent a complete psychological screening, including a formal evaluation to establish the presence of PTSD and to identify possible comorbid psychological disorders. Participants taking medication (e.g., tricyclic antidepressants, anxioloytics, painkillers) were not excluded. However, participants agreed (a) not to modify their medication (type or dose) 2 months prior to the start of the baseline assessments until the end of the post-test; (b) not to begin taking a psychotropic drug during the study, if possible.

The study was approved in January 2006 (#CER CRIR-138-0405) by the ethics committees at the Institut Universitaire en Santé Mentale de Montréal (Quebec) and the *Centre de Recherche Interdisciplinaire en Réadaptation* (CRIR) (Center for Interdisciplinary Research in Rehabilitation) with which the Centre de Réadaptation Lucie-Bruneau was affiliated. The same evaluator met with each participant at one center or the other, according to the origin of the referral.

### 2.5. Assessment

#### 2.5.1. Psychological Condition

The Structured Clinical Interview for DSM-IV–for Axis I (*SCID*; First, Spitzer [8]) was used to determine the primary and secondary diagnoses in the clinical groups. To establish the frequency and severity of PTSD symptoms in the PTSD and mTBI groups, a psychologist specialized in trauma administered the revised Clinician-Administered PTSD Scale for DSM-IV (CAPS; Blake, Weathers [9]). All events were civilian events. Motor vehicle accidents were the main target event for the two mTBI groups (75%), followed by assault (15%) and work accident (10%). However physical assault was the main target event for the PTSD group (78%), followed by work accident (14%) and vehicle accident (10%). The Beck Depression Inventory (BDI-II; Beck, Steer [10]) was used to evaluate the intensity of depressive symptoms. The impact of state and trait anxiety on cognitive performance was measured using the State-Trait Anxiety Inventory (STAI; Spielberger, Gorsuch [11]). All of the questionnaires described above have demonstrated good psychometric properties.

#### 2.5.2. Neuropsychological Evaluation

Following the psychological evaluation, all participants were given a battery of standardized neuropsychological tests. Readers are referred to a compendium of neuropsychological tests (e.g., Lezak [12]) for a detailed description of the psychometric properties of all of the instruments described below.

*Baseline measures of intellectual and perceptual integrity*. An analogical reasoning test (*Raven’s Progressive Matrices–Short Form*) and a summary exam of visuoperceptual functions were used to measure basic intellectual functioning and to ensure that basic perceptual functions were intact.

*Measures of attention capacity.* Immediate recall of a series of numbers (*Digit Span of* WAIS-III) and visuospatial sequences (*Corsi Blocks Task*) were used as span tasks. The dependent variables of these measures were the longest correctly recalled sequences both in order and in reverse order. A letter and symbol cancellation test (*Mesulam’s Cancellation Test*) was used to evaluate selective visual attention in a scanning task. The dependent variable on this measure was the time to complete the test. Among the tests of working memory, an adapted version of the *Stroop* sustained attention and inhibition task [13] was used to provide a more challenging test in terms of recruitment of attentional resources. The *Stroop* test has four subtasks (*Name, Colour, Interference, and Flexibility*). Task execution time and number of errors produced constituted the dependent variables for this task. A second working memory task was based on the *Brown-Peterson* paradigm; the task required the participant to remember a series of trigrams during a concurrent backwards counting task; participants were subsequently tested on their recall at different delay intervals (9, 18, and 36 s). The outcome measure used in the present study was the total number of correctly remembered letters at each delay interval. Finally, we used a rapid symbol substitution test (*WAIS-III Digit Symbol Test*) to evaluate divided attention in a visuographic context; the outcome measure was the total number of correctly substituted symbols within 120 s.

*Measures of learning capacity and long-term memory.* The *California Verbal Learning Test* *(CVLT-II*) [14] was used to measure long-term verbal learning. The CVLT-II involves repeated learning of a grocery list of items from four categories, with free or cued recall, followed by the presentation of a new list of words. Learning was measured by differed recall of the first list following a 20-min delay. Long-term visual memory was evaluated using the *Rey Complex Figure*. Participants were shown a figure and asked to reproduce it. They were subsequently tested on immediate and differed recall of the figure.

*Measures of executive functions.* The *Tower of London Test* was used to evaluate participants’ capacity to conceptualize, develop and execute the sequential movement of a series of beads (on three pegs) to reproduce different goal arrangements. Finally, we included a test of verbal fluidity (*Verbal Fluency*) developed by Delis, Kaplan [14].

In an effort to minimize the effect of fatigue on the cognitive test results, we reversed the order of the tests for half of the participants in each group.

## 3. Results

Descriptive and parametric statistics were calculated using SPSS-version 15. When the distribution of pooled data scores did not respect the assumptions of normality, score transformations (square roots or logarithms) were performed. Questionnaires with over 10% missing data were excluded from analyses. Missing data was replaced with the mean substitution method.

### 3.1. Primary and Secondary Psychological Diagnoses

The proportion of participants suffering from a PTSD diagnosis specified as moderate and severe (as assessed by the SCID-I) was greater in the PTSD group (64%) than in the mTBI/PTSD group (17%). Furthermore, 32% of participants in the PTSD group reported severe PTSD diagnosis, while none did in the mTBI/PTSD group. In the latter group, the majority of participants presented mild and moderate PTSD diagnosis (33%). Also, participants presented sub-clinical symptoms (33%) or partial PTSD (17%) in the mTBI group. In contrast, only 4% of participants in the PTSD group presented sub-clinical at the time of the evaluation. Based on Mylle and Maes [15] recommendations, the “sub-clinical” PTSD category includes the cases which did not reach the number of symptoms required for criterion C (avoidance) or D (neurovegetative hyperactivity), although at least one symptom of every criterion was present. The second category indicated by the term “partial” PTSD refers to the cases where one or another of the criteria is missing (intrusion and hyper-awakening) in spite of the significant presence of the F criterion.

Results on the SCID revealed that the PTSD group presented more secondary diagnoses than did the mTBI group. Secondary diagnoses reported by participants in the PTSD group included mood and anxiety disorders (panic disorder; social or specific phobias). In the mTBI/PTSD group, two participants presented current symptoms of depression and two others were in remission. One participant reported a current anxiety disorder in this group. In comparison, in the mTBI group, three participants with depression in remission, one participant with dysthymia, and two participants with remitted panic or generalized anxiety disorder were identified.

### 3.2. Control and Clinical Variables

Significant differences in group means on control and clinical variables are reported in Table 1.

**Table 1:** Summary of Control and Clinical Variables.

Sociodemograp Variables	PTSD (*n* = 25)	mTBI (*n* = 19)	mTBI/PTSD (*n* = 6)	Controls (*n* = 25)	*F (df)*	Part η*^2^*
Age	*M (SD)* 38.5 (12.4)	*M (SD)* 40.3 (14.7)	*M (SD)* 33.3 (15.9)	*M (SD)* 38.9 (12.6)		ns
Female ^a^ Education	19/25 (76%) 14.8 (3.1)	9/19 (47.4%) 13.4 (3.9)	2/6 (33.3%) 12.8 (3.1)	19/25 (76%) 15.4 (2.6)		ns
Elapsed time (in months)	48.5 (41.8) (range 2–146)	30.7 (31.6) (3–98)	9.0 (2.9) (4–11)	N/A	*F* (2, 47) 3.44 *	0.13
Clinical	*M (SD)*	*M (SD)*	*M (SD)*	*M (SD)*	*F* (3, 65)	
Beck-II	29.8 ^b^ (13.0)	11.6 ^d^ (11.4)	23.8 ^b^ (10.6)	4.8 ^b^ (5.0)	25.81 **	0.54
STAI State	54.0 ^c^ (11.0)	37.6 ^c^ (12.9)	43.3 (7.8)	29.3 (6.3)	*F* (3, 67) 25.65 **	0.53
Trait	59.5 ^c^ (10.2)	41.1 ^c^ (14.3)	55.8 (8.6)	32.6 (10.4)	25.18 **	0.53

^a^ Female/male ratio was not equivalent across groups (Fisher’s exact test, *p* = 0.047); Due to missing data, *n* was 1 ^b^, 2 ^c^ or 3 ^d^ lower; * *p* < 0.05; ** *p* < 0.01.

Significant differences between groups were observed for ratio of female to male participants. The mTBI/PTSD group had significantly less time elapsed since the trauma than the PTSD group. Post-hoc analyses revealed that the individuals in the PTSD and mTBI/PTSD groups were significantly more depressed and anxious than the individuals in the control group and the mTBI group. The PTSD group differed significantly from the mTBI group on anxiety dimensions, while no differences in anxiety, or in measures of mood disorder, were found between the mTBI group and the controls.

### 3.3. Neuropsychological Test Results

Table 2 presents the main results of the ANOVAs for each group on each of the neuropsychological test administered. Only the significant between-group results are described in greater detail.

**Table 2:** Summary of Clinical Group Results on all Neuropsychological Tasks.

Tasks	PTSD (*n* = 25)	mTBI (*n* = 19)	mTBI/PTSD (*n* = 6)
Attention			
Span	Normal	Normal	Normal
Cancellation	Normal	Normal	Normal
Brown-Peterson	Normal	Normal	Normal
Stroop			
Speed	Deficit ^1^	Deficit ^2^	Deficit ^3^
Errors	Deficit ^1^	Normal	Deficit ^4^
Digit Symbol	Deficit	Deficit	Normal
Long-term Memory			
Visual (Rey Figure)	Normal	Normal	Normal
Verbal (CVLT-II)	Normal	Normal	Deficit ^5^
Executive Functions			
Verbal Fluency	Deficit ^6^	Deficit ^7^	Normal
Tower of London	Normal	Normal	Normal

Legend: Normal = results not significantly different from Controls. ^1^ = deficit in all task conditions; ^2^ = deficit in the Colour condition only; ^3^ = deficit in Name and Colour conditions only; ^4^ = deficit in all conditions, except corrected errors more frequent in Interference and Flexibility conditions; ^5^ = deficit in Differed free and cued recall and Recognition conditions; ^6^ = deficit in Letter and Category conditions; ^7^ = deficit in Letter condition only.

### 3.4. Stroop Task

The conditions in which a major group effect was observed are identified in Table 3.

Analysis of variance revealed a significant group effect on the *Stroop* task. Post-hoc analyses demonstrated that the three clinical groups were significantly slower than the control group on the basic color naming condition (*Colour*). The PTSD and mTBI/PTSD groups were also slower than the control group on the reading condition *(Name)*. On *Interference* and *Flexibility* conditions, the PTSD group response times were significantly slower than those observed in the control group; the mTBI and mTBI/PTSD groups did not demonstrate significantly greater susceptibility to distraction than did the control group.

**Table 3:** Completion Time in Seconds on Stroop Task (Raw Scores).

	Means and Standard Deviations for Each Condition					
	PTSD	mTBI	mTBI/PTSD	Control		
Condition	*M* (*SD*)	*M* (*SD*)	*M* (*SD*)	*M* (*SD*)	*F ^a^* (3, 74)	Part. η*^2^*
Name	49.5 (8.3)	46.5 (11.9)	50.8 (5.3)	39.7 (5.2)	8.18 **	0.26
Colour	74.0 (14.5)	66.4 (12.4)	73.8 (11.7)	55.4 (8.5)	12.61 **	0.35
Interference	135.4 (47.2)	114.5 (25.1)	120.0 (16.1)	102.6 (47.6)	4.96 *	0.17
Flexibility	150.3 (59.5)	128.1 (25.8)	128.8 (16.6)	106.2 (21.0)	8.20 **	0.26

^a^ *F* was calculated on logarithmic transformations of raw scores; * *p* < 0.05; ** *p* < 0.01.

An analysis of variance on the transformed (as per Bohnen, Twijnstra [13]) response time scores was conducted to determine whether the slowness observed in the PTSD group on the *Interference* and *Flexibility* conditions was attributable to the participants’ observed slowness in the basic conditions. This calculation weighed the participants’ response times on the *Interference* and *Flexibility* conditions as a function of their response times on the *Name* and *Colour* baseline conditions. The results confirm that the significant differences observed between the PTSD group and the control group in the *Interference* (F (1, 48) = 4.57, *p*<0.05, η^2^ = 0.09) and *Flexibility* (F (1, 48) = 11.14, *p*<0.05, η^2^^=^ 0.19) conditions were maintained when participants’ response times were weighed based on their respective base levels.

A descriptive analysis of the total errors and the most frequently committed errors observed in each group was conducted to determine whether slower execution time was partially a function of number of errors. Types of error include corrected errors (participant self-corrects immediately, increasing his or her total time) and uncorrected errors (participant does not notice the error).

*Total errors*. The results revealed that 16% of participants in the control and mTBI groups made more than eight errors in total, while over 52% and 50% of participants in the PTSD and mTBI/PTSD groups, respectively, committed as many errors.

*Corrected errors.* Sixty percent of participants in the PTSD group and 50% of participants in the mTBI/PTSD groups committed 6–14 corrected errors; the proportion of participants in the mTBI and in the control groups committed 6–14 errors were 16% and 24%, respectively. Further, corrected errors occurred most frequently in the *Interference* conditions. The proportion of PTSD and mTBI/PTSD group participants who committed 6–14 corrected errors in the combined I*nterference* and *Flexibility* conditions were 40% and 33%, respectively; in contrast, the proportions in the mTBI and control groups were 10% and 4%, respectively.

*Uncorrected errors*. The mTBI/PTSD group had the highest proportion (33%) of participants who made more than 5 uncorrected errors. The proportions in the PTSD, mTBI, and control groups were 12%, 10.6%, and 4%, respectively.

### 3.5. Digit Symbol Task

Significant slowing was observed in the clinical groups on the visuographic task ((*F*(3, 74) = 4.56, *p* < 0.05, η^2^ = 0.16)). Post-hoc analyses confirmed significant differences in means between the PTSD group (*M* = 68.9, *SD* = 13.5), the mTBI group (*M* = 68.7, *SD* = 18.9), and the control group (*M* = 81.6, *SD* = 12.6).

### 3.6. California Verbal Learning Test

Results on the various recall conditions of the *CVLT* verbal learning test are reported in Table 4.

**Table 4:** Results on California Verbal Learning Task (CVLT-II).

Groups Var.	PTSD M (SD)	mTBI M (SD)	mTBI/PTSD M (SD)	Control M (SD)	F (3, 74)	Part η2
Total 1–5 (A)	59.6 (10.2)	55.4 (13.4)	50.0 (17.0)	62.2 (11.0)	2.33	0.09
Imm. FR (B)	7.8 (2.6)	6.4 (2.7)	5.3 (2.0)	7.9 (2.4)	2.79 ^a^	0.11
Imm. FR (A)	12.4 (2.9)	11.8 (3.9)	10.5 (6.2)	13.4 (2.8)	1.42	0.06
Imm. CR (A)	13.1 (2.4)	12.5 (3.5)	11.0 (5.0)	14.1 (2.5)	2.08	0.08
Diff. FR (A)	12.7 (3.1)	12.3 (3.7)	9.3 * (5.4)	14.0 (2.9)	3.15 *	0.12
Diff. CR (A)	13.1 (2.7)	12.9 (3.3)	10.5 * (5.0)	14.4 (2.2)	3.23 *	0.12
Pro. Interfer.	−0.64 (2.1)	−0.94 (3.1)	−0.67 (1.4)	−0.64 (2.0)	0.07	0.00
Retro Interfer.	1.7 (1.9)	0.72 (2.5)	1.7 (3.1)	1.2 (1.4)	0.87	0.04
Recognition	14.8 (1.4)	14.6 (2.0)	12.8 * (3.5)	15.4 (1.0)	3.82 *	0.14

Variables: *Total 1–5 (A)* = Sum of items from list A recalled correctly after five trials; *Imm. FR (B)* = Immediate free recall of items from list B; *Imm. FR (A)* = Immediate free recall of items from list A after free recall of list B; *Imm. CR (A)* = Immediate cued recall of items from list A; *Diff. FR* (A) = Differed free recall of items from list A; Diff. CR (A) = Differed cued recall of items from List A; *Pro. Interfer.* = Proactive interference (*i.e.*, sum of items recalled from list B minus sum of items recalled from trial 1 of list A; *Retro. Interfer*. = Retroactive interference (*i.e.*, sum of items recalled correctly from Imm. FR (A) minus sum of items from trial 5 of list A; *Recognition =* Forced choice selection of presented items among non-presented distracters; * *p* < 0.05 for main and post-hoc effects ^a^ *p* reached 0.05 significance criteria for main effect of group, but post-hoc comparison was not significant.

Significant main effects in group were found for the following conditions: *Immediate free recall of list B*, *Delayed free/cued recall,* and *Recognition of list A*. Post-hoc analyses revealed that only the mTBI/PTSD group had significantly weaker means than the control group, and only in the *Delayed recall* conditions *(i.e., Free recall, Cued recall,* and *Recognition)*.

### 3.7. Verbal Fluency Task

The main effects of group and the mean scores on the *Verbal Fluency* test are reported in Table 5.

**Table 5:** Verbal Fluency Task Scores.

	Means and Standard Deviations for Each Condition					
Groups	PTSD	mTBI	mTBI/PTSD	Control		
Condit.	*M* (*SD*)	*M* (*SD*)	*M* (*SD*)	*M* (*SD*)	*F (3, 74)*	Part. η*^2^*
Letters	29.3 (7.5)	35.0 (9.5)	31.8(9.9)	43.6 (9.9)	10.82 *	0.31
Category	37.4 (6.8)	39.5 (8.1)	39.5 (7.6)	45.9 (8.7)	5.26 *	0.18
Alternation	12.1 (3.4)	13.8 (3.9)	10.9 (5.4)	14.2 (3.6)	2.23	0.09

* * p* < 0.05.

The results on the measure of verbal fluidity indicated a more marked slowing in evocation mechanisms for verbal long-term memory in the PTSD group, in both the lexical and categorical recall conditions. In the mTBI group, slowing was limited to the lexical evocation task, which requires greater attentional control.

## 4. Discussion

At first glance, the results seem to confirm the present study’s first hypothesis, in that greater cognitive deficits were observed in the PTSD and mTBI/PTSD groups than in the control group. The results suggest that the PTSD group had significantly greater deficits on some measures of divided attention and attentional interference than did the mTBI group. However, this finding is mitigated by the significant comorbidity with depressive and anxious symptoms observed in the PTSD group; the presence of comorbid symptoms limits the plausibility of the unique contribution of PTSD to the results. Therefore, the first two hypotheses are only partially confirmed.

The third hypothesis seems to be confirmed, at least partially, by the findings; only the mTBI/PTSD group presented problems with verbal long-term memory, distractibility and divided attention. Like the PTSD group, the mTBI/PTSD group committed more corrected errors than the mTBI group on the attentional inhibition test, particularly in the context of attentional interference. However, participants in the mTBI/PTSD group presented only mild to sub-clinical PTSD symptoms in the majority of cases; therefore, the presence of PTSD cannot entirely explain the more severe verbal long term memory problems observed in the mTBI/PTSD group than in the PTSD group. The participants in the PTSD group presented moderate to severe symptoms of post-traumatic stress in the majority of cases, but did not present deficits in long-term memory. Furthermore, given that the intensity of depressive and anxious symptoms was comparable between the mTBI/PTSD and PTSD groups, comorbidity alone cannot account for the more marked deficits in long-term memory observed in the former group. Therefore, how can we explain that the verbal long-term memory deficit is observed in the comorbid PTSD and mTBI group but not in the other two clinical groups?

One reasonable explanation for the results obtained is as follows: the combination of PTSD symptoms, significant anxiety, and depression symptoms contributes to deficits in divided attention, deficits in categorical and lexical fluidity, and greater distractibility, without creating problems related to long-term memory. It appears that when an mTBI is “added” to PTSD (even mild PTSD) with significant comorbid depression and anxiety, an interaction effect occurs between the mTBI and the combined psychological conditions (PTSD, depression, and anxiety), exacerbating cognitive problems to the point of affecting long-term memory, rather than affecting attention only. This explanation contradicts the hypothesis of a “cumulative effect” of the respective impacts of mTBI and PTSD, suggested by Vanderploeg, Belanger [5]. The interaction effect is further supported by the fact that the participants in the mTBI group, who did not present trauma symptoms or comorbid depression or anxiety, also presented no difficulties with verbal learning, despite neurological diagnostic criteria equivalent to that of the mTBI/PTSD group. Neurophysiologically, we could hypothesize that subtle neurological or functional deficits attributable to mTBI create a “disinhibition” effect in fronto-temporal regulation mechanisms [16,17,18,19]. This could result in greater difficulty in regulation of PTSD symptoms in individuals with mTBI because attentional resources are still limited [18,20]. This hypothesis could account for the greater disturbances in attention and memory processes observed in the sample mTBI/PTSD than in the sample with mTBI without psychological comorbidity. In sum, the negative interaction between neurophysiological and/or functional effects of mTBI and psychological comorbidity may produce a multiplicative rather than additive negative effect on cognitive performance.

An alternative explanation for the results obtained may be the shorter period of elapsed time since the trauma in the mTBI/PTSD group (9 months) than in the PTSD group (48.5 months). This difference may account for the more severe cognitive problems observed in the former group. In fact, it is theoretically possible that the individuals in the mTBI/PTSD group were still in a state of neurological recovery at the time of the study, or still reacting to the sequelae of mTBI, resulting in more significant psychological distress than that observed in the individuals in the mTBI group. Future studies with larger samples of participants with dual diagnoses will help determine the relevance of the amount of elapsed time since the trauma.

The clinical data obtained in the present study seems to suggest that a diagnosis of PTSD, with or without concomitant mTBI, is associated with increased comorbid anxious and depressive symptoms. The differences between groups in the intensity of trauma symptoms constitutes another interesting result; moderate to severe trauma symptoms were observed in the PTSD group, whereas the symptoms observed in the mTBI/PTSD group were mild to subclinical. The results also demonstrate the need to develop diagnostic tools for clinical populations which present both post-traumatic symptoms and symptoms of neuropsychological conditions such as mTBI simultaneously [21]; such instruments would improve differential diagnosis. The fact that 50%–60% of participants recruited in the PTSD and mTBI groups were excluded from the study confirms the need to control for comorbidity and sociodemographic factors in these populations in order to avoid reports of cognitive deficits attributable to conditions other than PTSD and mTBI.

The results obtained in the PTSD group are consistent with the results of Isaac *et al.*’s [1] literature review of studies of PTSD populations. In particular, we found an absence of deficits on measures of attention that used more proceduralized mechanisms (e.g., *Span tasks, Letter/Symbol cancellation*), in comparison to more complex and challenging tests that demand greater attentional resources (e.g., *Stroop, Symbol Digit*). The results of the present study are also congruent with those reported by Nelson, Yoash-Gantz [6] in a study of individuals with comorbid PTSD and mTBI. However, the significant depressive and anxious comorbidity reported by individuals in the PTSD group in the present study may have contributed to the observed cognitive difficulties. The specific contribution of PTSD to attentional deficits and problems in delayed verbal recall in some groups of PTSD cannot be definitely confirmed.

In their meta-analysis of significant neuropsychological sequelae in mTBI populations, Belanger, Curtiss [3] concluded that sequelae from mTBI were generally observed in clinical samples rather than in population-based samples, and were limited to the acute medical phase (less than three months post-event).

The authors found that neuropsychological sequelae primarily affected verbal fluidity and delayed verbal recall. Given that post-concussive and post-traumatic symptoms in the mTBI/PTSD group in the present study were chronic rather than acute, the similarity between Bélanger *et al.*’s [3] results and the results of the dual diagnosis group in the present study are surprising. This finding raises the question of control over comorbid factors, and the impact that such control or lack thereof may have had on the results of several studies described by Bélanger and colleagues. Finally, the authors mentioned that some of the results in the mTBI samples included in the review may have been attributable to psychological factors; however, meta-analysis methods did not control these variables [22,23].

One of the primary methodological limitations of the present study was the number of participants; the limited sample size prevents generalization of the results. Another limitation was the clinical nature of the samples; individuals in clinical samples had either been treated or were seeking treatment at the time of recruitment, and were not representative of general (non-clinical) populations of individuals with PTSD and mTBI. The use of clinical questionnaires that had not been validated with all the populations recruited in this study further highlights the need to develop sensitive and specific instruments for PTSD and mTBI populations.

The results of the present study have several clinical implications. First, concerning the diagnosis, the results confirmed the importance of using structured interviews rather than self-report questionnaires to diagnose PTSD in mTBI populations. Given the overlap in symptoms between mTBI and PTSD, as well as the tangling of symptoms in the case of a comorbid PTSD and mTBI, the use of this questionnaire as a diagnostic tool poses the risk of false-positive diagnoses of PTSD in mTBI populations [24]. Second, the results allow us to identify several neuropsychological characteristics specific to each clinical group. Neuropsychological profiling is an interesting avenue for future research and will allow clinicians to look beyond the common symptoms between the conditions and to identify the specific cognitive and behavioral problems of each population. Such profiling may be particularly relevant for differential diagnosis.

## 5. Conclusions

The results obtained on the standardized measures of attention, memory and executive functions suggest a more marked sensitivity to attentional distraction in the PTSD group than in the mTBI group. Only the mTBI/PTSD group, however, demonstrated problems in verbal long-term memory, in addition to attention problems of a comparable nature and intensity to those observed in the PTSD group. However, given the weaker intensity of PTSD symptoms in the mTBI/PTSD group, trauma symptoms cannot entirely account for the results of the memory tests, any more than can comorbidity considered in isolation. The possibility of an interaction between subtle sequelae of mTBI and emotional symptoms (PTSD, depression, mixed) is suggested as an alternative explanation for the results presented here. A second alternative explanation is the greater recency of the trauma in the mTBI/PTSD group than in the PTSD or mTBI groups. These results highlight the importance of closely controlling comorbid factors and time elapsed since trauma in studies of the psychological impacts of trauma on cognitive performance. The results also demonstrate the utility of documenting the cognitive profiles of different subgroups of individuals with PTSD. Establishing specific neuropsychological profiles for subgroups could promote accurate differential diagnosis and the development of effective therapeutic interventions for clinical subgroups with similar symptoms.
